# Willingness to Pay for Healthy Housing During the COVID-19 Pandemic in China: Evidence From Eye-Tracking Experiment

**DOI:** 10.3389/fpubh.2022.855671

**Published:** 2022-03-15

**Authors:** Xiaotong Guo, Zhaoyang Fan, Hong Zhu, Xiangyang Chen, Mengmeng Wang, Hanliang Fu

**Affiliations:** ^1^School of Management, Xi'an University of Architecture and Technology, Xi'an, China; ^2^Laboratory of Neuromanagement in Engineering, Xi'an University of Architecture and Technology, Xi'an, China; ^3^School of Marxism, Xi'an University of Architecture and Technology, Xi'an, China

**Keywords:** willingness to pay, healthy housing, information attentiveness, eye tracking, COVID-19 pandemic, theory of planned behavior

## Abstract

Healthy housing can set its occupants completely in good physical, mental and social conditions, but there is a lack of research in China on the public's willingness to pay (WTP) for healthy housing. From the perspective of cognitive psychology, this study constructs an analytical framework based on the model of “theory of planned behavior” (TPB), the theory of selective information exposure, and the model of “emotions as social information,” while exploring the effect mechanism of the online reviews on the public's WTP for healthy housing during COVID-19 pandemic. In combination with eye-tracking experiments and subjective reports, physiological, psychological and behavioral multimodal data on WTP of 65 participants for healthy housing are collected. Partial least squares structural equation modeling (PLS-SEM) is adopted to analyze the formation effect mechanism of the public's WTP for healthy housing. This study acquires the following results: (i) Information attentiveness to online reviews on different valence information of healthy housing as obtained in eye tracking experiments delivers significant effect on attitude, subjective norm (SN) and perceived behavioral control (PBC), but has no direct effect on the public's WTP for healthy housing; (ii) Hypotheses from TPB model are verified. attitude, PBC and SN can all make significant effect on WTP for healthy housing, with attitude showcasing the most prominent effect; and (iii) In terms of the mediating effect, information attentiveness can deliver significant indirect effect on WTP through attitude.

## Introduction

The World Health Organization (WHO) announced the outbreak of the highly transmittable coronavirus (COVID-19) as a pandemic in March 2020 (1). Although this outbreak is expected to be improved in the next few years, the COVID-19 pandemic has undoubtedly imposed broad impact on society and consumers, resulting in big changes in individual's willingness to consume (2). Since the outbreak of COVID-19, people from more and more countries are learning and working at home. Especially, during the isolation period, the house would become the only place for people to spend most of their time every day. Over the past decade, plenty of scientific evidence has demonstrated that all aspects of the built environment would exert far-reaching and directly measurable impact on physical and mental health outcomes at all population levels (3). Therefore, the completeness and healthy levels of various living facilities and their influences on human health are particularly important, especially when people are now becoming more concerned about their health because of the outbreak of COVID-19. A 2021 survey by Accenture on more than 3,000 consumers across 15 countries illustrates that the pandemic is likely to create a more sustainable and healthier consumption era within the following 10 years (4). Combined with new transformations, the existing efforts are to benefit built environments and all inhabitants, to better prepare humans for the COVID-19 and other similar crises that may occur in the future, and to enhance resilience of communities. Living in a healthy housing environment can effectively improve the health status of individuals and enhance immunity levels, which would help contain the spread of COVID-19 as well as its harm to human body.

WHO defines healthy housing in this way: on the basis of meeting the basic requirements of housing, highlighting the health elements, with the concept of sustainable development of human living health, to meet the physiological, psychological and social needs of residents, and to create a healthy, safe, comfortable and environmentally friendly high-quality residential region and community. In other words, healthy housing should be able to make the occupants live in a physically, mentally and socially good condition completely. However, in developing countries such as China, the public's requirements for the scale of housing space have just been met, and citizens are currently in the initial stage of improving their quality of living (5). In fact, most of the people live in ordinary housing, which does not provide such facilities as fresh air systems or constant temperature and humidity systems that are equipped with healthy housing (6). In the initial stage of novel technology's diffusion or new product's promotion, the public often holds some cognitive biases (7). Such factors as perceived usefulness, price, technology readiness and public opinion may all play a negative role in the sales promotion of healthy housing to consumers in China. Given that WTP is an important indicator to predict the promotion of healthy housing, it is particularly critical to explore the main factors and means that affect the public's WTP for healthy housing.

China's healthy housing industry started late: it was not until 2016 that the Architectural Society of China had issued the first version of “Assessment Standard for Healthy Building” (T/ASC02-2016). So far, the total area of buildings with healthy building labels cross the whole China is about 10 million square meters, while in just one city, e.g., Xi'an, there are more than 400 million square meters of various types of residential buildings. These data indicate that the market penetration of healthy housing in China is still limited. It can be seen that healthy housing is indeed a new type of residence building in China. Increasing the public's demand and willingness to pay (WTP) for healthy housing will not only help enhance the physical and mental health condition of the whole people, but would also promote the development of the healthy housing industry effectively. The research topic of this study includes two aspects: healthy housing, and WTP for housing consumption. Over recent decades, increasingly more studies have been carried out on the relationship between urban architectural environment and human health in indoor and outdoor space (8). Specifically, the existing studies on healthy housing are closely related to public health issues, such as tuberculosis care (9) and carcinogenic risks of indoor organic contaminants to residents (10). Scholars have also evaluated exposure to drifting secondhand smoke in multiunit housing for young adults, in addition to the elderly and sick (11). Or in the case of shared housing, the intersectional impacts of stranger dangers on young adults' health and wellbeing are explored (12). The WTP for housing consumption is a traditional research field, and its currently hot research topics include: the consumption attractiveness of green housing (13), the demand characteristics of sustainable housing (14), the preference of young adults for shared housing, and so on (15). There are similarities between healthy housing construction standards and the green or sustainable housing, but differences in public perceptions of them directly lead to distinctions in consumer behavior. For example, green residential consumption is a pro-environmental behavior and is altruistic, while healthy housing consumption is essentially driven by egoism and is a purchase behavior triggered by one's own health needs. Therefore, some studies use health awareness or health facilities as one of the variables to explore the formation path of housing consumption intention. Wan et al. (16) obtained that the main factor that promotes consumer's WTP for green furniture constantly is health awareness by use of factor analysis and logistic regression. Won et al. (17) explored the correlation between the health level of residential environment and the occupancy rate of public rental housing. Wu et al. (18) analyzed the rural resident's WTP for and participate in the improvement of rural sanitation facilities. In general, few previous studies have directly taken the public's WTP for healthy housing as their main research topic. As mentioned earlier, recent studies investigating the influence of the COVID-19 pandemic on living consumption have shown that consumers are increasingly concerned about the health functionality of products (19). Therefore, it is necessary to adjust classical theoretical models by introducing important and new factors that may affect healthy housing consumption, so as to better explain the public's WTP for healthy housing during the COVID-19 pandemic.

In the dynamic process of making consumption decisions, consumers will perform a lot of information retrieval actions. For new products, especially for those made in novel technology, the vast majority of people, including acquaintances, have not used. “See what others have to comment” becomes a key factor in decision-making. On housing, a kind of high-value consumer products, the public opinion has a greater impact, especially for Chinese people, who are more willing to make housing consumption decisions that conform to the public's preference, due to their common personality traits, such as cautiousness and compliance. With high popularity of information technology, social media become the main way for people to obtain public opinion. The former provides relatively stable interpersonal relationships and is prone to normative influences, while the latter offers relatively variable relationships and generally has informational influences (20). This study will consider the influence of public opinion on WTP for healthy housing in a wider interpersonal community of strangers, build up a social review scene for information intervention, and uses an eye-tracking system to record the participant's eye movement trajectories. Then, their pupil diameter, fixation time and other indicators are used to measure the attention process of online reviews. Commonly used in experimental equipment for psychological and medical research, eye trackers have, over recent years, got gradually increased application in consumer research for analyzing food types, packaging, label design, advertisements, supermarket shelves, food menus, and consumption selection process of other visible information (21). However, it is rare to carry out eye-tracking experiments in housing consumption-related research. Sun et al. (22) used eye-tracking experiments to simulate the real online housing renting process. Hollander et al. (23) explored how the hidden brain design directs individual engagement with the built environment by using eye-tracking emulation software. Therefore, under the background of people's increasing concern about healthy life caused by COVID-19 pandemic, this study proposes an extended model in “theory of planned behavior” (TPB) and designs an eye-tracking experiment and questionnaire survey to explore the influencing mechanism on the public's WTP for healthy housing from the perspective of cognitive psychology. Specifically, this study attempts to explore the following research questions: First, what factors affect the public's WTP for healthy housing? Second, are there interactions among the variables? And third, what is the impact path of online reviews on the public's WTP for healthy housing? With a focus on the research gap of previous studies on the consumption intention of healthy housing, this study will provide certain reasonable suggestions on the promotion of healthy housing.

The remainder of the study is organized as follows: Section Theoretical background and hypotheses explains the theoretical framework and demonstrates the research hypotheses. Section Materials and methods illustrates the eye tracking experiment design. Section Results presents results and analysis. Section Discussion includes discussion on research findings. Finally, Section Conclusion provides conclusions, policy suggestion, and study limitations.

## Theoretical Background and Hypotheses

### Theoretical Background

To explore the effect of the public's health awareness and attitude toward health on their acceptance of healthy housing, it is necessary to understand the psychological mechanism behind individuals' behavior intention. The conceptual framework of this study is founded on TPB, which is purposed to explain individuals' behavior more reasonably (24). In the TPB model, the public's willingness to accept healthy housing depends on a number of predictors, including attitude toward healthy housing, subjective norm (SN) about healthy housing consumption, and perceived behavioral control (PBC) over healthy housing. Although TPB has been widely adopted in the existing literature, some non-negligible limitations exist in its application. Therefore, various refinements and extensions of TPB theory have been put forward to improve its effectiveness and applicability. For example, researchers added such variables as health consciousness (25) and perceived consumer effectiveness (26) to the TPB model.

Recently, many studies have applied the TPB model to analyze consumer's green purchasing behavior. For example, Hsu et al. used the TPB model to investigate individual's behavioral intention to visit green hotels and found that attitude, SN and PBC affect behavioral intention positively (27). Zahan et al. (28) consider the green purchase intention of Bangladeshi consumers toward Green Housing through augmented TPB implantation. According to individual loss aversion psychology in prospect theory, the difference between loss aversion and WTP has a significant correlation. Decisions about healthy housing consumption depend on whether the public pays for the welfare of increasing personal health.

The Web 2.0 specification enables people to collaborate and share information with others over the Internet. Social media have become one of the most influential marketing tools for companies to strengthen communication with customers, because they cannot only provide a platform for sharing personal life, but also become an important consumer communication tool to affect all aspects of consumer behavior, including information acquisition, attitude, consumption, and product service evaluation (29). Information on social platforms will affect consumers' impression of it, and providing information through social media has attracted more and more public attention (30). Because the public opinion received from social platforms has higher reliability than that from traditional methods, such as questionnaire surveys, this study adds the variable of information attentiveness to the TPB model to explore the effect of social media on the public's WTP for healthy housing.

### Research Hypotheses

#### Information Attentiveness

Communication scholars have used eye-tracking methods for decades, for example, to study individual's attention to advertising (31). During the COVID-19 pandemic, social media have become one of the mainstream ways for people to obtain information under indoor isolation. In other words, through social media, people communicate with each other and obtain the information they need. Social platforms focus people's attention on some hot topics. Under the influence of the COVID-19, people pay more and more attention to health issues, so the topic of healthy housing has also gradually attracted more attention. Public opinion on healthy housing as showcased on social media can better reflect the public's attitude toward healthy housing. The mainstream social media platforms in China include Weibo, WeChat, Baidu Tieba, Zhihu and so on, through which the public can search for information about healthy housing. And such information may affect the public's WTP for healthy housing. Yu et al. (32) found that external affective events interact with individual's decision-making by using biological, physiological, and neuroscientific tools.

In the process of receiving information, individuals always consciously or unconsciously choose to accept information that is the same as their own opinion, while avoiding information that goes against theirs. This process is called “selective exposure to information,” which is prevalent in consumers' purchase scenarios (33). When an individual's overall evaluation of a stimulus target is positive, he/she will be more willing to receive and process positive information; vice versa. Similarly, selective exposure to information affects individual's attention to public opinion on healthy housing, and the fixation duration for different valences of public reviews will be different.

The Emotions as Social Information Model (EASI) aims to explain how others' emotions affect an observer's decision-making through affective response and inference processing mechanisms, as well as the moderating effects of cognitive motivation and appropriateness judgment (34). The public opinions on healthy housing in social media are individual opinions with their unique emotions. When reading a stimulus page, participants will take their emotions brought by these online reviews as part of the information, thus affecting their individual judgment and cognition (35). Therefore, this study defines information attentiveness as the average fixation time of participants to different valences of public opinions on healthy housing, with the corresponding study hypotheses discussed below.

H1a: Information attentiveness to different online review valence about healthy housing has a significant and direct effect on attitude.
H1b: Information attentiveness to different online review valence about healthy housing has a significant and direct effect on subjective norm.
H1c: Information attentiveness to different online review valence about healthy housing has a significant and direct effect on perceived behavioral control.
H1d: Information attentiveness to different online review valence about healthy housing has a significant and direct effect on WTP for healthy housing.

#### The Relationship Between Individual Perceptions and WTP

Attitude refers to an individual's positive or negative evaluation of certain behavioral outcomes. The existing studies have found that attitude can reflect consumer's psychological evaluation of products, thus delivering a significant effect on consumers' behavioral intention (36). Based on the TPB model, some recent studies have identified health-related consumption intentions and behaviors and demonstrated that the outcome expectancy, the general attitude toward vaccines, and SN are the most important determinants of behavioral intentions in regard to influenza vaccination (37). Attitude represent individual's subjective opinions on things, so individuals' attitude toward healthy housing will affect their WTP for healthy housing. To this end, this study proposes the following hypothesis:

H2: Attitude has a significant and direct effect on WTP for healthy housing.

SN refers to the perceived social pressure which influences the execution or non-execution of any evident behavior. A number of studies have indicated that the perceived social pressure is an important factor in consumer behavior (38). For example, according to social norms, individuals with high SN prefer environmentally-friendly consumption and may have a significant indigenous effect on their consumption intention (39). Strong SN, intolerance, public stereotype and other factors all can affect WTP (40). Based on the above analyses, this study proposes the following hypothesis:

H3: Subjective norm has a significant and direct effect on WTP for healthy housing.

PBC refers to the potential difficulties when people conduct a particular behavior. In addition, studies have found that health awareness and environmental awareness affect consumption intention, and that PBC may deliver the greatest impact on such intention (41). In this study, PBC means that the public has time, money and ability to pay for their healthy housing. In other words, the more the public can control the above mentioned factors, the more the behavioral intention will be conducted (42). As such, the following hypothesis is proposed in this study:

H4: Perceived behavioral control has a significant and direct effect on WTP for healthy housing.

**Figure 1:**
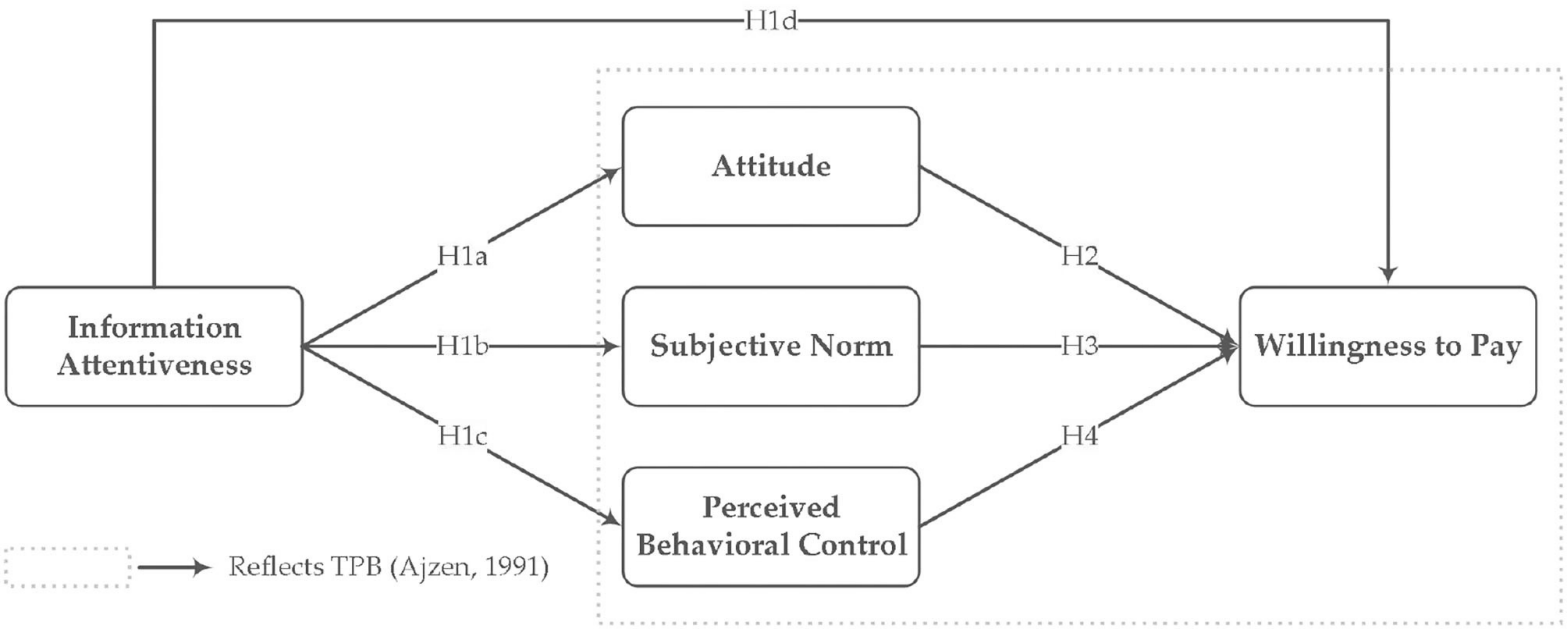
Conceptual model and hypotheses.

Based on the above mentioned theories and hypotheses, this study establishes a mechanism model of the effect of information attentiveness on public's WTP for healthy housing (Figure 1).

## Materials and Methods

### Participants

In order to determine the mechanism of online reviews on the public's consumption intention of healthy housing, this study combines questionnaire survey and eye tracking experiment. In the eye tracking experiment, a real social platform scene was simulated, and eye tracking sensor was used to capture the eye movement trajectory of participants when browsing the online review that whether they have WTP for healthy housing. After the eye tracking experiment, participants were asked to complete a questionnaire consisting of two parts: the first part is demographic, such as age, gender, education, health condition and so on; the second part is individual cognition of healthy housing, and all items were used Likert 7-level scale, of which 1 represents very opposed, and 7 represents very agreed. The questionnaire is designed according to the relevant literature and is improved in combination with the research topic in this study to ensure the validity and reliability (shown in Table 1).

**Table 1:** List of measurement items.

**Construct**	**Items**	**Reference**
Attitude (ATT)	(ATT1) I think healthy housing consumption is necessary.	(43)
	(ATT2) I think healthy housing consumption is a good idea.	(44)
	(ATT3)I think healthy housing is safe.	
	(ATT4) I think healthy housing has advantages over traditional housing.	
Subjective Norm (SN)	(SN1) My family thinks I should be spending on healthy housing rather than traditional housing.	(43)
	(SN2) My close friends think that I should be spending on healthy housing rather than traditional housing.	(45)
	(SN3) Most people who are important to me think that I should be spending on healthy housing rather than traditional housing.	
Perceived Behavioral Control (PBC)	(PBC1) I believe I can afford healthy housing over traditional housing whenever I want.	
	(PBC2) I see myself as capable of healthy housing consumption in the future.	(46)
	(PBC3) I have the resources, time and willingness to make healthy housing consumption.	(47)
WTP for healthy housing (WTP)	(WTP1) I would like to make healthy housing consumption.	(45)
	(WTP2) I would like to live in healthy housing.	(46)
	(WTP3) I would like to recommend healthy housing to my family and friends.	

Seventy participants in Xi'an were recruited to join the eye-tracking experiment. All participants are healthy, have normal vision or correct vision, have the ability to analyze things. Five participants were excluded for eye tracking data analysis because their data collection rates are <80%. Finally, 65 valid samples were obtained. After completing the complete experiment, the participants were rewarded with CNY 45 in cash or a gift of the same value. The laboratory of neuromanagement in engineering of Xi'an University of architecture and technology approved this study.

Table 2 shows the demographic characteristics. There were 65 participants, including 32 males (49.2%) and 33 females (50.8%). The ratio of male to female is almost equal, which can avoid the effect of gender on experiment results. Because this experiment was carried out in the Laboratory of Neuroengineering Management, Xi'an University of Architecture and Technology, the 20–30 age group has the highest proportion of all participant (81.5%). Among all the participants, the proportion of bachelor's degree was the highest (56.9 %), followed by master's degree and above (38.5%). The participants' educational level is relatively high. In addition, about 80 % of the participants are in good long-term health, and about 90 % of their family members are in good long-term health.

**Table 2:** Demographic characteristics of the participants (*N* = 65).

**Description**	**Items**	**Frequency (percentage)**
Gender	Male	32 (49.2)
	Female	33 (50.8)
Age	20 or less	6 (9.3)
	20–30	53 (81.5)
	30–40	3 (4.6)
	40 or more	3 (4.6)
Education	High school and below	1 (1.5)
	Specialist college	2 (3.1)
	Undergraduate degree	37 (56.9)
	Master's degree and higher	25 (38.5)
Health conditions	Physical health	51 (78.5)
	Current recovery from	2 (3.0)
	past medical history	
	Self-feeling sub-health	12 (18.5)
Health status of	Physical health	57 (87.7)
family members	Chronic diseases	6 (9.2)
	In-hospital treatment	2 (3.1)

### Stimulus Materials

**Figure 2:**
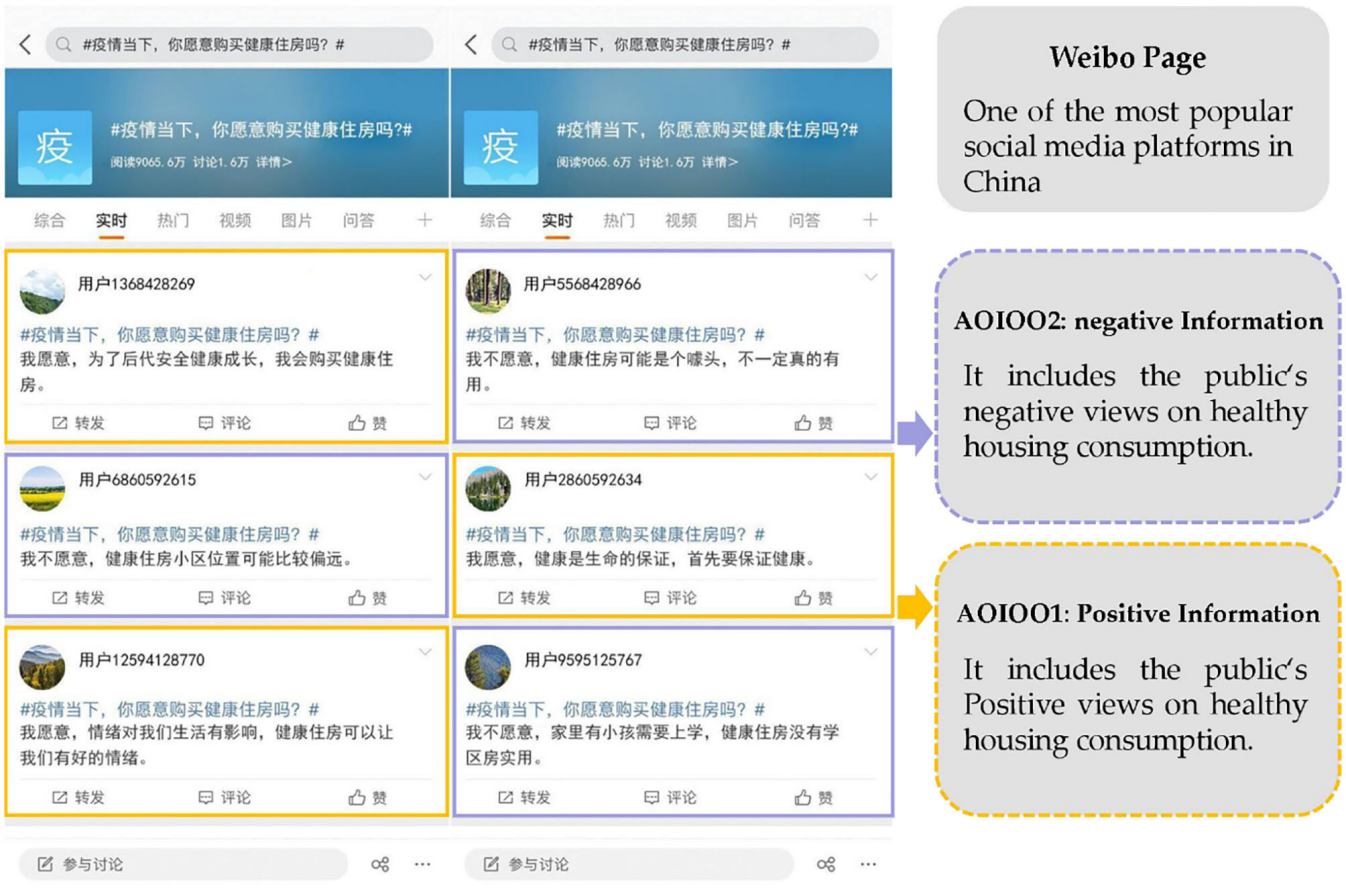
Area of interest division map of Weibo page.

Sina-Weibo (http://us.Weibo.com), often referred to as Weibo, is the most popular social media platform in China. Weibo had over 530 million active users each month in 2021. In order to observe and record participants' online search behavior for healthy housing reviews, this study used Weibo forum page to present eye-tracking stimulus materials pictures which contain different valence online reviews on healthy housing. This experiment set the online reivew area to two AOIs (Areas of Interest), positive reviews (AOI001) and negative reviews (AOI002). In order to avoid the disturbance of reading order on the experimental results, each forum page contains 3 AOI001 and 3 AOI002 respectively. The order of AOIs is random and the character count of review is basically the same. AOI001 refers views why some Weibo users are willing to consume healthy housing, while AOI002 highlights the views why others are unwilling to consume healthy housing. The stimulus material is derived from real online review on healthy housing in Weibo, excluding some extreme review. Considering that the duration of fixation may be caused by the ease of semantic comprehension, this study selected online review with clear semantic representation, the character count of each page was about 270 words. In order to avoid the disturbance of social media profile photo on this experiment, it was all replaced with a landscape image. The stimulation page is shown in Figure 2. The fixation duration of different AOIs can explain participants' cognitive activity and information attentiveness.

### Experimental Facilities and Experiment Procedure

In this study, the equipment is Tobii Pro Fusion model eye monitor. The eye tracker connected directly to a 15.6-inch laptop via USB Type-C with a resolution of 1920 × 1080. The eye tracker uses near infrared light to produce reflective images on the cornea and pupil of user's eyes, and then uses two image sensors to collect images of the eye and the reflection. Finally, the image processing algorithm and a three-dimensional eyeball model are used to accurately calculate the position line of sight of the eyes in space. During the eye tracking experiment, slight head movement has little effect on the accuracy of eye tracking data. Participants did not need to wear the device during the experiment just watch the laptop screen.

Eyes move quickly when reading, and the individual's attention is shifted from one part of the stimulus material to another. Then when the brain is in information processing stage, the eyes remain almost unchanged. Thus, eye-tracking method assumes that there is a direct relationship between individuals' fixation and what they are focusing on. Researchers set AOIs on some parts of the stimulus material, analyze the eye tracking that occur in these areas. The longer average fixation duration in an AOI, indicating that it is more important and attractive to the participant than other AOIs. By comparing the fixation duration participants spent on AOIs, Fu et al. (48) justify that consumer's online reviews search behavior is substantially affected by human contact degrees of recycled products. Therefore, in this study, the average fixation duration was used to represent the participants' interest in AOIs.

**Figure 3:**
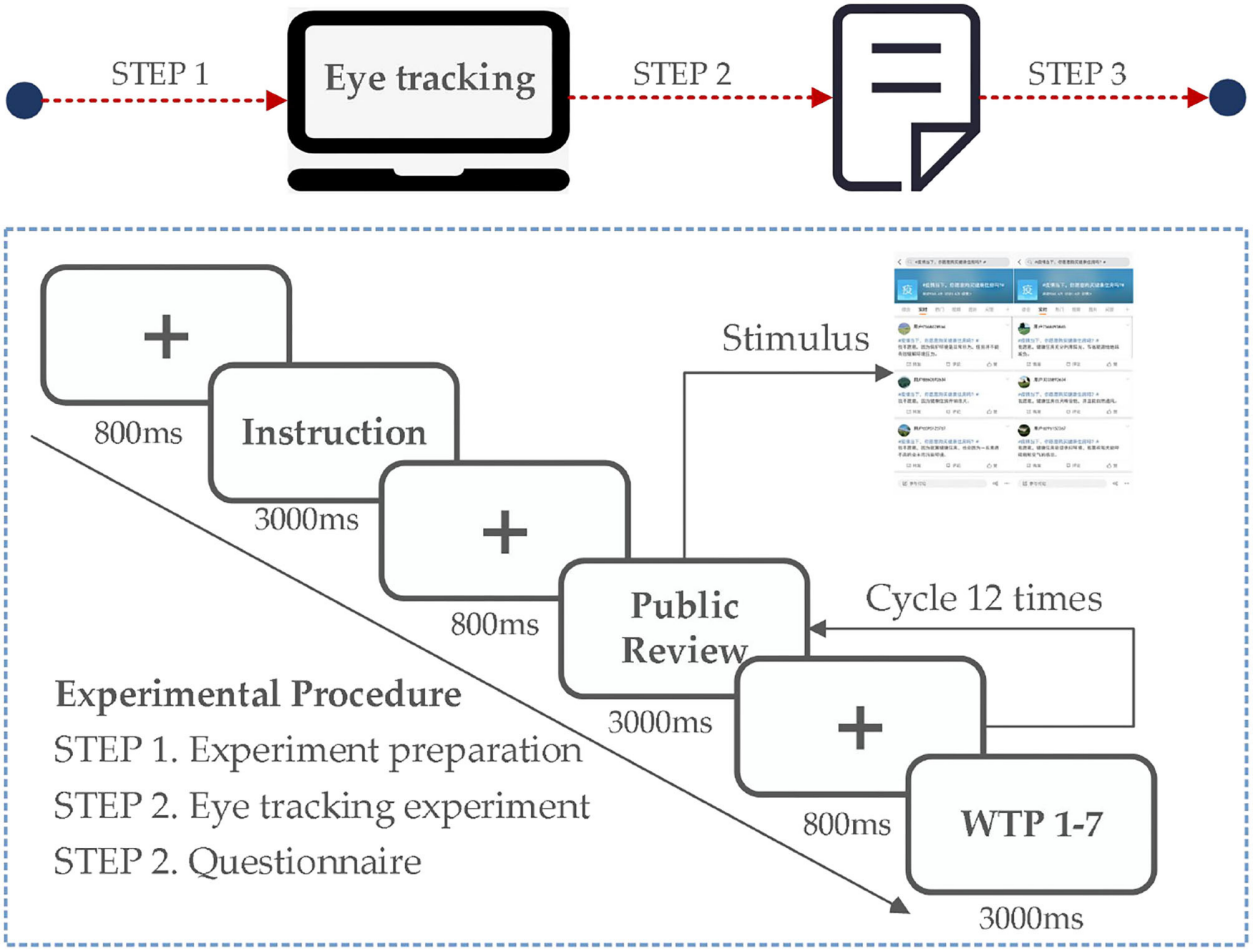
Experimental procedures.

Participants were asked to choose a comfortable sitting position to better complete the experiment. The distance between the eye tracker and the participant's eyes should be maintained at 60–65 cm, with the line of sight in the center of the screen. Five-point calibration will be carried out before the beginning of the experiment, which contains accuracy and precision. Accuracy and precision should be controlled below 0.05° as far as possible. In order to avoid differences in participants' understanding of healthy housing, an introduction page will be presented to the participants before the formal experiment begins. The first page is an experimental introduction. After reading the introduction page, the participants press the space bar to begin the formal eye tracking experiment. In order to ensure that the participants can browse the entire stimulus completely, college students were recruited for the pre-experiment, and the presentation time of the stimulus was finally determined to be 30 s. The eye tracking experiment consists of 12 trails with a total duration of about 10 minutes, and the stimulus materials present randomly. After experiment, the participants were asked to fill out a questionnaire. Before the formal questionnaire survey, a pilot study was conducted to test the reliability and validity of the scale items. Considering the complexity of the eye tracking experiment and the minimum sample size requirement of PLS-SEM model, 20 college students were recruited to participate in the eye tracking experiment and questionnaire. Cronbach's alpha correlation test and principal component analysis (PCA) were used to analyze the reliability and validity of the survey items in the pilot study. In the reliability examination, the Cronbach's alpha coefficient of potential variables was between 0.765 and 1, which was greater than the threshold value. In the validity examination, the KMO value of potential variables was greater than the threshold value 0.5, and the *P*-value of the Bartlett test of potential variables was lower than 0.05. The results of the factor analysis indicated that the absolute value of the loading coefficient of the observed variables on the factors they relate to was between 0.543 and 0.781, greater than the threshold value 0.5. Therefore, the samples have good reliability and validity, and the data meet the qualification of the examinations. The data collection process is shown in Figure 3.

### Data Analysis

Tobii Pro Lab software was used for preliminary processing of eye tracking data. Formula (1) was used to calculate the information attentiveness of participants to online review about healthy housing. *IA* refers to information attentiveness, *FT*_AOI001_ refers to average fixation duration of AOI001 (positive review), and *FT*_AOI002_ refers to average duration time of AOI001 (negative review). The information attentiveness <0.5 means that the participants pay more attention to the negative review, and the information attentiveness >0.5 means that the participants pay more attention to the positive review.


(1) $$\begin{array}{r} IA\;=\;{FT}_{\text{AOI}001}/\left({FT}_{\text{AOI}001}\;+\;{FT}_{\text{AOI}002}\right) \end{array}$$

An extended TPB model was propose in this study, partial least squares structural equation modeling (PLS-SEM) was adopted to examine the effect mechanism of public opinion on the public's WTP for healthy housing. PLS-SEM is a regression-based approach that minimizes the residual variances of the endogenous constructs (49). As measures were developed with a Likert scale, data have a non-normal data distribution. PLS does not require any normality assumptions and handles non-normal distributions relatively well (50). General sample size for PLS is between 30 and 100, which is more than ten times the count of construct. The sample size of this study was 65, and the count of construct was 5, which met the requirements of PLS. This study uses Smartpls 3.0 software as a common analysis software for partial least squares path modeling, and uses the bootstrapping method of 5,000 resampling to evaluate the statistical significance of path coefficients.

## Results

### Descriptive Statistics

Discriminant validity was examined by comparing the square root of AVE and the correlation coefficients between variables. The results are shown in Table 3. The square root of a variable's AVE should be higher than the correlation coefficients involving that variable (51). Attitude (ATT), PBC and SN were positively related to WTP. All the square root of AVE was higher than the involving correlation coefficients, indicating that the discriminant validity of each construct was acceptable.

**Table 3:** Discriminant validity.

**Construct**	**Mean**	**SD**	**VA**	**ATT**	**PBC**	**SN**	**WTP**
VA	0.51	0.03	**1.000**				
ATT	4.94	1.46	0.493	**0.835**			
PBC	4.97	1.33	0.489	0.636	**0.799**		
SN	3.96	1.12	0.264	0.460	0.461	**0.889**	
WTP	4.72	1.23	0.471	0.780	0.674	0.602	**0.913**

*IA, information attentiveness; ATT, attitude; PBC, perceived behavioral control; SN, subjective norm; WTP, willingness to pay*.

### Reliability and Validity

The quality of the measurement model was assessed by reliability and validity testing. Reliability was tested with Cronbach's alpha value (52). Table 4 presents the results from the reliability and validity analysis of each measurement. All of the Cronbach's alpha values were higher than the threshold of 0.7. Hence, it can be concluded that the measurement model had good reliability. In regard to convergent validity, all variables presented high composite reliability (CR), with scores ranging from 0.840 to 0.919, which were above the recommended standard of 0.6. Additionally, the values of AVE (0.638 to 0.791) were above the acceptable limit of 0.5. Therefore, the convergent validity of the measurements was satisfied.

**Table 4:** Reliability and validity analysis.

**Construct and items**	**Factor load**	**Cronbach alpha (CA)**	**Composite reliability (CR)**	**Average variance extraction (AVE)**
**Attitude (ATT)**		0.856	0.901	0.697
ATT1	0.343			
ATT2	0.345			
ATT3	0.247			
ATT4	0.254			
**Subjective Norm (SN)**		0.868	0.919	0.791
SN1	0.410			
SN2	0.345			
SN3	0.369			
**Perceived Behavioral Control (PBC)**		0.716	0.840	0.638
PBC1	0.360			
PBC2	0.482			
PBC3	0.405			
**Willingness to Pay (WTP)**		0.900	0.937	0.833
WTP1	0.388			
WTP2	0.343			
WTP3	0.364			
**Information Attentiveness (IA)**	1.000	1.000	1.000	1.000

Additionally, the discriminant validity was tested with the Heterotrait–Monotrait (HTMT) ratio through the application of the PLS algorithm (53). The HTMT is defined as the mean value of the item correlations across constructs relative to the (geometric) mean of the average correlations of the items measuring the same construct. When constructs are conceptually similar, HTMT between two variables is generally required to be <0.85.It can be seen from Table 5 that Most of the HTMT values between the model variables in this study are <0.85 (only one indicators being >0.85). Thus, evidence of both convergent and discriminant validity was found.

**Table 5:** Heterotrait-monotrait ratio.

**Construct**	**IA**	**ATT**	**PBC**	**SN**	**WTP**
IA	—	—	—	—	—
ATT	0.523	—	—	—	—
PBC	0.571	0.796	—	—	—
SN	0.285	0.512	0.576	—	—
WTP	0.495	0.873	0.833	0.673	—

### Model Verification

In this study, the boostrapping method with 5,000 re-sampling times is used to verify the hypothesis of causal relationship between latent variables, and the statistical significance of path coefficients is evaluated by calculating the T-Statistics of each path coefficient. The results are shown in Table 6. First, the direct effects of the independent variables on the dependent variables were examined. Information attentiveness has direct and positive effects on ATT, SN, and PBC, so H1a, H1b and H1c are supported. However, the relationship between information attentiveness and intention to consume healthy housing was not significant, so H1d is not supported. Most the independent variables were found to have significant effects on the dependent variables.

**Table 6:** Results for the hypothesis model.

**Hypothesis**	**Path**	**Path coefficient**	***t-*Value**	***P-*value**	**Results**
H1a	IA → ATT	0.493	4.864	0.000	Yes
H1b	IA → SN	0.264	2.059	0.037	Yes
H1c	IA → PBC	0.489	4.179	0.000	Yes
H1d	IA → WTP	0.052	0.638	0.527	No
H2	ATT → WTP	0.501	4.889	0.000	Yes
H3	SN → WTP	0.260	3.763	0.000	Yes
H4	PBC → WTP	0.211	2.373	0.010	Yes

The direct effects of the independent variables on the mediators and the effect of the mediators on the dependent variables were examined. Standardized coefficient estimate points indicated that the paths between ATT and WTP, SN and WTP, PBC and WTP and were significant and positive, so H2, H3, and H4 are supported. Information attentiveness has significant effects on all individual perception variables, and the three individual perception variables have significant effects on WTP. Hence, we assumed that ATT, PBC and SN may have mediating effects between information attentiveness and WTP. We used bootstrap method to exam the significance of the indirect effects. The results show that information attentiveness has a significant indirect effect on WTP through ATT (*p* < 0.05). However, information attentiveness has no significant indirect effect on WTP through PBC and SN (*p* > 0.05). Table 6 shows the standardized path coefficients, *t*-values, and results.

**Figure 4:**
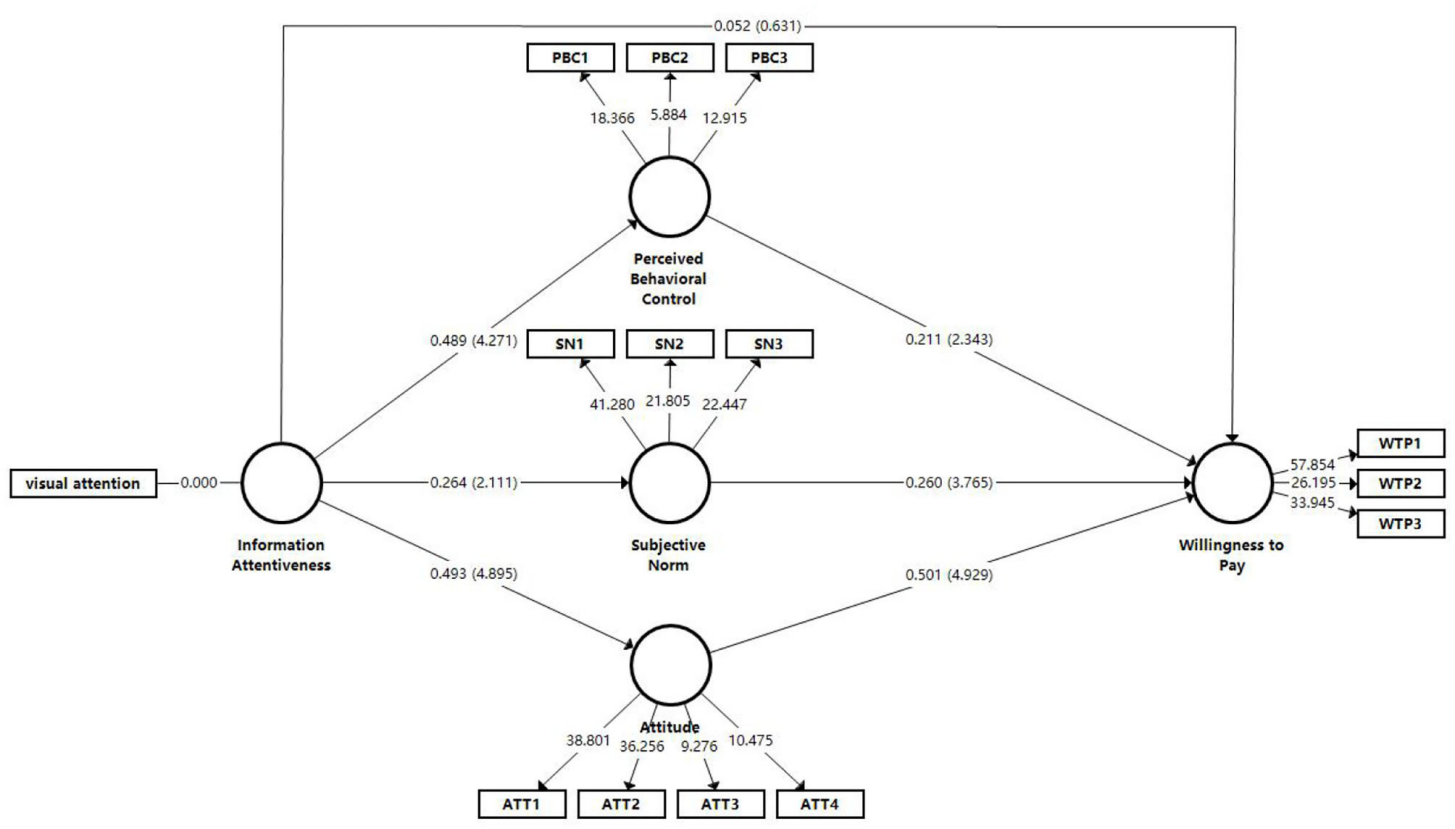
Model path for WTP for healthy housing.

The results show that the direct effect of ATT on WTP is higher than PBC and SN. The effect of information attentiveness on ATT is greater than the effect on PBC than on SN. Figure 4 shows the path coefficient and effect.

### Goodness of Fit

Tenenhaus et al. (54) proposed a diagnostic tool as the goodness of fit (GoF) index of PLS-SEM for evaluating model fitting. This GoF is measured by using the geometric mean value of the average communality score (AVE values) and the average R^2^ values (for endogenous constructs) and is calculated using following equation [GoF = √(AVE × R^2^)]. According to Henseler et al. (55) a good model fit indicates that a model is parsimonious and plausible. Thus, GoF index was calculated for the model involved in this study, which is presented in Table 7. As shown in the table below, the GoF index of the conceptual model used in this study is 0.639, indicating that the GoF model fits very well. The results show that the theoretical model proposed in this study has significant predictive correlation and explanatory power.

**Table 7:** Calculation of GoF index.

**Constructs**	**AVE**	***R^2^***
Attention	1.000	
Attitude	0.485	
Perceived Behavioral Control	0.283	
Subjective Norm	0.545	
Willingness to Pay	0.626	0.695
Average scores	0.588	0.695
AVE * R^2^	0.409	
GoF = √(AVE × R^2^)	0.639	

## Discussion

Many previous studies have explored the effect of attitude, PBC and SN on the WTP for housing consumption, but they have generally failed to conduct research on the WTP for healthy housing under COVID-19. Although examining the moderating effects of attitude, PBC and SN on these relationships, such researchers have not explored the mediating effect in these relationships. In addition, the effect of public opinion on WTP for healthy housing is yet to be discussed. Therefore, this study proposes an extended TPB model to explore the effecting mechanism of the public's information attentiveness on WTP for healthy housing during COVID-19. The results verify the importance of information attentiveness, attitude, SN and PBC to Chinese people' WTP for healthy housing under COVID-19.

Firstly, the public's information attentiveness to online reviews on different valence information of healthy housing obtained through eye-tracking experiments is found having strong influence on attitude, SN and PBC. The public's information attentiveness to the different valence information of healthy housing imposes no direct effect on WTP. This finding is not completely consistent with the results of previous studies. Han et al. (56) found that the daily frequent discussion on Weibo is synchronized with the trend of the COVID-19 pandemic in the real world. Nowadays, the public is very sensitive to the pandemic and major social events. The difference between the results of this study and of Han et al. may be due to many factors affecting the WTP for healthy housing. Household wealth will be taken into account when people make housing consumption. However, the empirical result of this study shows that there is only one mediating effect “information attentiveness → attitude → WTP.” When the public gets positive information on healthy housing from social media, their health awareness will be enhanced, meaning that they care about health. While the public further understands healthy housing, they will gradually show a positive attitude toward healthy housing consumption. The results demonstrate that information attentiveness can affect WTP through the mediating effect of attitude. What can be proved is that social media play a vital role in guiding the public's WTP for healthy housing. After obtaining useful information through social media, the public would pay further more attention to the positive information in social media, indicating that they are more interested in the positive information on healthy housing, which will encourage them to build a stronger attitude toward healthy housing. Therefore, the government can publicize and promote healthy housing through various social media, so that people can benefit from healthy housing. The COVID-19 pandemic are pushing up consumers' demand for more space and less dense living environments. In the post-pandemic era, new requirements for housing are bound to emerge. It is not difficult to observe from this study's results that due to the prevalence of COVID-19, housing selection has undergone major changes. People prefer healthy housing because it is good for human's wellbeing and health (57). And the results also show that there is no high popularity of healthy housing, and the concept is not clear enough. In order to improve the WTP for healthy housing, it is necessary to boost related concepts of healthy housing among the public.

Secondly, attitude deliver the most significant and positive effect on WTP for healthy housing. Although with a positive attitude toward healthy housing, the public's WTP is often tempted by their attitude, so they may choose to spend more on healthy housing. This finding is consistent with the discoveries about the general consumption of the products that are beneficial to human health (58). The more the public knows about healthy housing, the higher their attitude toward healthy housing consumption and the more attention to health needs; as a result, the WTP for healthy housing will become stronger. This study finds that SN has a relatively weak relationship with intention in the TPB model, a result consistent with the literature that has taken TPB as the underlying theory. SN refers to the effect of social environment on the public's WTP for healthy housing. A weak and insignificant relationship is identified between SN and WTP. The reason for such relationship may be the small sample size of this study and the relationship between housing consumption structures. Because the housing prices are relatively high, people will consider their own financial situation. PBC of healthy housing has a positive but not strong effect on WTP, meaning that the public has enough knowledge on different aspects in using healthy housing information, thus prompting them to change their traditional behavior by promoting their consumption intention (59). This finding is consistent with that of previous studies. Some studies have explained that this phenomenon is related to the current status of housing consumerism in China (60). On the one hand, it is necessary for couples to purchase a house when getting married. On the other hand, with the rapid development of urbanization, housing prices are rising sharply year after year. So it is very difficult for young adults to purchase their own houses. Instead, most young adults need parental help in housing consumption. Therefore, young adults' PBC has little effect on WTP for healthy housing.

At last, as for additional constructs, demographics results show that the average WTP for healthy housing is 4.717, with 62 participants (95%) being highly educated, indicating that the education level is also related to the public's WTP for healthy housing. The higher the education level, the clearer the concept of healthy housing and the stronger the health awareness. In general, under the background of COVID-19, the public's WTP for healthy housing is still kept at a high level. Under the COVID-19 pandemic, people are paying increasingly more attention to their own health and healthy housing, possibly because people are forced to spend long time indoors and their thermal comfort and overall comfort must remain. In addition, gender factors are not reflected in this study.

## Conclusion

This study tries to explore the influencing mechanism on the public's WTP for healthy housing in China under COVID-19 pandemic. By considering the effect of public opinions on WTP for healthy housing, this study proposes an extended TPB model. Based on eye-tracking experiments and subjective report data, the PLS-SEM is used to explore the cause-effect relationship among the influential factors on WTP for healthy housing. Moreover, with all the measurement scales employed, the proposed model in the study is also confirmed to be appropriate. The main conclusions are as follows: (i) Attitude, PBC and SN have significant effect on public WTP for healthy housing, and the effect of attitude is the most significant. (ii) The public's information attentiveness to the online review on different valence information of healthy housing obtained through eye tracking experiment had strong influence on attitude, SN and PBC. (iii) The public information attentiveness to the different valence information of healthy housing has no direct effect on WTP for healthy housing. (iv) In terms of mediating effect, the public's information attentiveness has a significant indirect effect on WTP for healthy housing through attitude.

Based on the results above, this study proposes the following suggestions on how the government can improve the public's WTP for healthy housing. First, the public's health awareness and pandemic awareness have a direct effect on their attitude toward healthy housing. Due to their limited understanding of the use of healthy housing, the public instinctively believes that healthy housing has no specialties compared with ordinary housing. Therefore, it is necessary to strengthen the concept of universal healthy housing and the importance of healthy housing during the pandemic prevention and control period. Specifically, policy makers should use social media to inform the public what is healthy housing by circulating targeted information (quality, advantages, price-performance, etc.). And the dissemination of information can be particularly targeted at individuals with low awareness of the pandemic and health. In addition, high prices of healthy housing should be lowered to promote the public's WTP for healthy housing. Second, for many people, their houses are now the only place where they can work and spend free time in the COVID-19 pandemic. So it can be assumed that a new household preference in demands could emerge after this crisis, driving households to look for more healthy houses, since this asset will increase its importance in living and working. Relevant departments should speed up the development of health facilities and accelerate the construction of healthy housing. Without timely supply of healthy housing, it may not be possible to guide the public's attention and demand for it.

This study is not free from all kinds of shortcomings, which shall be considered if further study is conducted. On the one hand, the samples used in this study may limit the generalizability of the findings, because most participants are recruited from colleges and the sample demographics are high education levels and young ages. Although this study meets the sample size requirement both for eye-tracking research and statistical analysis, future studies might include participants with other characteristics, so as to address this limitation. On the other hand, this study only explores the WTP, but not the behavior of payment. However, the public's consumption intention does not guarantee their actual behavior. Therefore, future research needs to explore more models by adding consumption behavior. In addition, in the future, more advanced cognitive neurological experimental equipment such as EEG can also be used to obtain the implicit attitude of individuals more accurately (61).

## Data Availability Statement

The original contributions presented in the study are included in the article/supplementary material, further inquiries can be directed to the corresponding author.

## Ethics Statement

The studies involving human participants were reviewed and approved by Laboratory of Neuromanagement in Engineering, Xi'an University of Architecture and Technology. The patients/participants provided their written informed consent to participate in this study.

## Author Contributions

XG, ZF, XC, HZ, HF, and MW: conceptualization and writing—review and editing. XG, ZF, and XC: formal analysis, methodology, and writing—original draft. ZF and HZ: investigation. HF, HZ, and MW: supervision. All authors contributed to the article and approved the submitted version.

## Funding

The study was funded by the National Natural Science Foundation of China (Grant No. 72104192).

## Conflict of Interest

The authors declare that the research was conducted in the absence of any commercial or financial relationships that could be construed as a potential conflict of interest.

## Publisher's Note

All claims expressed in this article are solely those of the authors and do not necessarily represent those of their affiliated organizations, or those of the publisher, the editors and the reviewers. Any product that may be evaluated in this article, or claim that may be made by its manufacturer, is not guaranteed or endorsed by the publisher.
